# Viral Agents of Diarrhea in Young Children in Two Primary Health Centers in Edo State, Nigeria

**DOI:** 10.1155/2015/685821

**Published:** 2015-04-30

**Authors:** Paul Erhunmwunse Imade, Nosakhare Odeh Eghafona

**Affiliations:** ^1^Department of Medical Microbiology, University of Benin Teaching Hospital, PMB 1111, Benin City 300283, Nigeria; ^2^Department of Microbiology, Faculty of Life Sciences, University of Benin, PMB 1154, Benin City 300283, Nigeria

## Abstract

Enteric viruses have been shown to be responsible for diarrhea among children during their early childhood. This study was carried out to determine the prevalence of rotavirus, adenovirus, and norovirus infection in young children with diarrhea in two primary health centers in Edo State, Nigeria. A total of 223 stool specimens were collected from children aged 0–36 months with clinical signs of diarrhea and 59 apparently healthy age-matched children as control. These specimens were investigated for three viral agents using immunochromatographic technique (ICT). The overall results showed that at least one viral agent was detected in 95/223 (42.6%) of the children with diarrhea while the control had none. The prevalence of rotavirus was 28.3%, adenovirus 19.3%, and norovirus 3.6%. There was a significant association between age group and infection (*P* < 0.0001). Seasonal pattern of enteric viruses was not statistically significant (*P* = 0.17). The overall coinfection rate was 7.6% and rotavirus-adenovirus coinfection had the highest with 5.4%. Rotavirus was the most prevalent viral agent. Coinfections are not uncommon among the population studied. The most commonly associated clinical symptom of viral diarrhea in this study was vomiting. Viral diagnostic tests are advocated for primary health care facilities in this locality.

## 1. Introduction

Enteric viruses are major etiologic agents of acute gastroenteritis among infants and young children worldwide. Rotavirus, norovirus, adenovirus, and astrovirus are the recognized viral causes of pediatric gastroenteritis [1]. The World Health Organization (WHO) data showed that each child practically has viral diarrhea irrespective of race and socioeconomic status within the first 5 years of life and this has great economic burden for the system of public health services and all society [2, 3]. Viral intestinal infections are the most common cause of acute infectious diarrhea in the pediatric group and accounted for approximately 70% of episodes of acute infectious diarrhea in children. Viral infections affect small bowel enterocytes and cause low grade fever and watery diarrhea [4].

There is paucity of information as regards viral enteropathogens of diarrhea in many developing countries including Nigeria. Many hospitals in Nigeria do not carry out routine diagnostic tests for viral diarrhea. Investigations on diarrheal infections are usually based on bacterial and parasitic agents. The reason(s) attributed to this may be as a result of the poor health care system in the country where important health issues are taken for granted such as the aforementioned case. Thus, as a result of the nonperformance of viral tests for diarrheal patients, information on viral enteropathogens is lost and thus measures to implement control strategies become difficult. Against this background, this study was carried out to ascertain the prevalence of rotavirus, adenovirus, and norovirus infection in young children with diarrhea in two primary health centers in Edo State, Nigeria. It also aimed at assessing the coinfection rates of the viral agents, age, seasonal distribution of infection, and the association between clinical symptoms and viral diarrhea.

## 2. Patients and Methods

### 2.1. Study Area

This study was carried out in two primary health centers located in Ikpoba-Okha Local Government Area of Edo State, Nigeria. The health centers attend to the primary health needs of the people within and around the locality. Cases attended to include malaria, diarrhea, immunization of infants and children, antenatal and postnatal cases, and other minor health issues within the scope of the health personnel. The two primary health centers are Aduwawa and Evbomodu primary health centers and they are neighboring communities with a fast growing population made up of indigenes with new residents from the main city alongside other inhabitants from other parts of the country.

### 2.2. Study Population

A total of two hundred and eighty-two (282) stool specimens comprising 223 diarrhea and 59 nondiarrhea stool specimens were collected from children aged between 0 and 36 months attending two primary health centers. As regards children with diarrhea, males were 121 while females were 102. Similarly, for the control, males were 33 while females were 26. Verbal informed consent was obtained from patients or guardian of the children prior to sample collection. Demographic and clinical information were obtained by means of structured questionnaire. The study was carried out from January 2011 to June 2012. Ethical approval was obtained from the local government health ethics committee.

### 2.3. Specimen Collection and Processing

The samples were collected from patients at the time of clinic visit as well as other times when the child defecates. Sterile wide mouth specimen containers were used for specimen collection and they were processed within 6 hours of collection. Rotavirus, adenovirus, and norovirus were detected by the immunochromatographic technique (ICT). Rotavirus and adenovirus were detected using VIKIA Rota-Adeno rapid test device manufactured by BioMerieux, France. Briefly, 2 drops of liquid stool specimen was added to the specimen dilution buffer and shaken vigorously to homogenize. Two drops of the diluted sample was transferred to the sample well of the test device (cassette) and was timed for 10 minutes. Results were interpreted according to the manufacturer's instructions. Similarly, norovirus was detected using RIDA Quick norovirus (NI403) test device manufactured by R-Biopharm AG, Germany. Briefly, 1 mL of sample dilution buffer (diluent) was placed in a separate labeled test tube and 100 mL of liquid stool was added to it and shaken vigorously to homogenize. This was allowed to settle for 2 minutes and 250 *μ*L of the supernatant was placed in a clean labeled test tube. Six drops of conjugate 1 was added to the test tube and was shaken vigorously to homogenize. The mixture was emptied into the sample well of the test device and incubated for 10 minutes at room temperature. Four drops of conjugate 2 was added to the reaction window of the test device and incubated for 1 minute at room temperature. 10 drops of wash buffer was added to the reaction window and was allowed to stay until the buffer was completely absorbed. Six drops of substrate was added to the reaction window and timed for 3 minutes. The results were interpreted according to the manufacturer's instructions.

Statistical analysis was carried out using odd ratio and chi-square (*χ* ^2^) tests. A *P* value of less than 0.05 was taken as significant.

## 3. Results and Discussion

A total of 223 children with diarrhea were tested for three viral agents (rotavirus, adenovirus, and norovirus). A total of 95 (42.6%) were positive for at least one viral agent while none of the 59 children without diarrhea was positive for any viral agent. The overall result showed that rotavirus had a prevalence of 63 (28.3%) (Table 1). The sex distribution of enteric viruses showed that males had 54 (44.6%) positive cases while females had 41 (40.2%), and this was not statistically significant (*P* = 0.60). Age group distribution of infection showed that 7–12 months had the highest infection rate with 48 (58.5%) and was closely followed by 0–6 months which had 32 (50.8%). There was a statistical significance between age group and infection (*P* < 0.0001) (Table 2). Monoinfections were 78 (35.0%) while coinfections were 17 (7.6%). The pattern of coinfecting viruses showed rotavirus-adenovirus mixed infection as the most prevalent with 12 (5.4%) (Table 3). The seasonal pattern of enteric viruses showed that rainy season had 60 (46.9%) while dry season had 35 (36.8%), and this was not statistically significant (*P* = 0.17). The distribution of enteric viruses according to health centers was not statistically significant (*P* = 0.89) (Table 4). The distribution of viral agents with respect to clinical symptoms is showed in Table 5.

The prevalence of rotavirus, adenovirus, and norovirus infections was investigated among children with diarrhea. The age range of the children was 0 to 36 months. The overall prevalence of viral agents was 42.6%. The results of this study when compared to other studies carried out in Nigeria and other parts of the world showed that the incidence of viral agents varied from one locality to another. The 28.3% of rotavirus in this study is lower than 55.9% in Ilorin, Nigeria [5], 35% in Jos, Nigeria [6], 33.3% in two districts in Nigeria [7], and 39% in Ghana [8]. It is higher than 27% in Zaria, Nigeria [9], 28.1% in Edo State, Nigeria [10], 9.2% in Botswana [11], and 16.9% in Korea [12]. The 19.3% of adenovirus in this study is lower than 22.3% in northwestern Nigeria [13] and 23.0% in Tanzania [14]. It is higher than 6.7% in Nigeria [7], 3.0% in South Africa [15], and 7.8% in Botswana [11]. The 3.6% of norovirus in this study is lower than 37.3% in Nigeria [16], 16.4% in Ghana [17], 39% in Brazil [18], 11.6% in Korea [12], and 15% in Ghana [19]. The differences among studies reporting viral infections in different countries might be explained by the different age group, seasonal variations, and viral detection methods used.

There was a statistical significance between infection and age group (*P* < 0.0001). Age group of 7–12 months had the highest prevalence of viral diarrhea with 48 (58.8%). This is consistent with the report of Moyo et al. [14], who also found 7–12 months as the group with the highest infection. The reason for this may be due to the fact that it is the period of activities for many children. Crawling and walking stages are at this period and in the process, children could pick harmful materials into their mouth especially in unhygienic conditions. It was observed in this study that, within the first year of life, viral infection rate was 84.2% and in the second year, it was 97.9%. Thus infections were more or less within the first two years of life. This is in accordance with other studies where infection was most prevalent in children within the first 2 years of life [11, 16, 19, 20]. The relatively low prevalence of viruses among older children could be partly due to immunity acquired through previous exposures [14].

In this study, coinfection rate was 7.6%, and rotavirus-adenovirus coinfection was the highest with 12 (5.4%). This is consistent with studies in Nigeria and Spain [13, 21]. This, however, did not agree with the reports of Chung et al. [12] and Koh et al. [22], who had rotavirus-norovirus mixed infection as the most prevalent.

The seasonal pattern of viral infection was not statistically significant (*P* = 0.17), though infection was more during the rainy season (46.9%) than in the dry season (36.8%). Reports from Nigeria have showed that viral diarrhea occurs throughout the year but with variations with respect to seasons [8, 13, 16]. This may be the reason for what was observed in this study. Viral diarrhea with respect to the two primary health centers did not show statistical significance (*P* = 0.89), as infection rates for Evbomodu and Aduwawa health centers were 43.0% and 42.1%, respectively.

The most commonly associated clinical symptom observed in this study with rotavirus, adenovirus, and norovirus positive cases was “vomiting” with 55.6%, 55.8%, and 75.0%, respectively. This finding is similar to other studies [23–25] where vomiting was the most commonly associated symptom with viral diarrhea. Rotavirus positive patients with respect to clinical symptoms such as fever, dehydration, and abdominal pain were low in this study compared to other studies [23, 26, 27] which reported high percentage of clinical symptoms. Similarly, for adenovirus positive patients, fever, dehydration, and abdominal pain were low compared to other studies with moderate symptoms [13, 24, 28, 29]. However, as regards norovirus positive patients, fever, dehydration, and abdominal pain were also low, but other studies with norovirus showed moderate to high clinical symptoms [25, 30, 31]. Thus, the differences in clinical symptoms with viral diarrhea as seen from the different studies may be attributed to seasonal variations, geographical location, nutritional status of the patients, and type of viral pathogens causing the infection.

## 4. Conclusion

In this study, viral agents associated with diarrhea were found to be 42.6%. Coinfection of viral agents was found to be 7.6% and the most common clinical symptom was vomiting. This is worrisome considering the burden of these viruses on the young children in this locality. The fact that viral diagnostic tests are not routinely done or rarely done in any of the hospitals in this locality means that such vital information on viral diarrhea is missed out due to the poor attention given to health care in the country. Thus, there is need to test stool specimens of clinically confirmed diarrheal patients for enteric viruses as this will go a long way in reducing the wasteful use of antibiotics which are used as blind treatment for persistent diarrhea that may be of viral origin. Finally, the primary health care centers should be provided with all the necessary diagnostic test materials to address cases of viral diarrhea.

## Figures and Tables

**Table 1:** Prevalence of three enteric viruses among children with diarrhea.

Enteropathogens	*n* = 223	Percentage
Number of children with at least one virus	95	42.6
Rotavirus	63	28.3
Adenovirus	43	19.3
Norovirus	8	3.6

**Table 2:** Sex and age distribution of viral enteropathogens.

Characteristics	Number tested	Number positive (%)	Rotavirus positive (%)	Adenovirus positive (%)	Norovirus positive (%)
Sex					
Male	121	54 (44.6)	36 (29.8)	19 (15.7)	5 (4.1)
Female	102	41 (40.2)	27 (26.5)	24 (23.5)	3 (2.9)
Total	** 223**	**95 (42.6)**	**63 (28.3)**	**43 (19.3)**	**8 (3.6)**

	*P* = 0.60				

Age group (months)					
0–6	63	32 (50.8)	20 (31.7)	14 (22.2)	2 (3.2)
7–12	82	48 (58.5)	31 (37.8)	23 (28.0)	3 (3.7)
13–18	35	8 (22.9)	7 (20.0)	3 (8.6)	2 (5.7)
19–24	26	5 (19.2)	4 (15.4)	2 (7.7)	1 (3.8)
25–30	11	2 (18.2)	1 (9.1)	1 (9.1)	0
31–36	6	0	0	0	0
Total	** 223**	**95 (42.6)**	**63 (28.3)**	**43 (19.3)**	**8 (3.6)**

	*P* < 0.0001				

**(a):** 

Characteristics	Number positive (%)	Rotavirus positive (%)	Adenovirus positive (%)	Norovirus positive (%)
Monoinfection	78 (35.0)	47	28	3
Coinfection	17 (7.6)	16	15	5
Total	**95** (**42.6**)	**63**	**43**	**8**

**(b):** 

Coinfection pattern	Number of patients	Percentage
Rotavirus + adenovirus	12	5.4
Rotavirus + norovirus	2	0.9
Adenovirus + norovirus	1	0.4
Rotavirus + adenovirus + norovirus	2	0.9
Total	**17**	**7.6**

**Table 4:** Seasonal pattern of enteric viruses and distribution according to health centers.

Characteristics	Number tested	Number positive (%)	Rotavirus positive (%)	Adenovirus positive (%)	Norovirus positive (%)
Season					
Dry	95	35 (36.8)	24 (25.3)	15 (15.8)	2 (2.1)
Rainy	128	60 (46.9)	39 (30.5)	28 (21.9)	6 (4.7)
Total	**223**	**95 (42.6)**	**63 (28.3)**	**43 (19.3)**	**8 (3.6)**

	*P* = 0.17				

Health centers					
Evbomodu	128	55 (43.0)	36 (28.1)	23 (18.0)	5 (3.9)
Aduwawa	95	40 (42.1)	27 (28.4)	20 (21.1)	3 (3.2)
Total	**223**	**95 (42.6)**	**63 (28.3)**	**43 (19.3)**	**8 (3.6)**

	*P* = 0.89				

**Table 5:** Distribution of rotavirus, adenovirus, and norovirus positive patients by some variables.

Variables	Rotavirus positive *n* (%)	Adenovirus positive *n* (%)	Norovirus positive *n* (%)	Total *n* (%)
Fever				
Yes	21 (33.3)	13 (30.2)	1 (12.5)	35 (30.7)
No	42 (66.7)	30 (69.8)	7 (87.5)	79 (69.3)
Total	**63 (100.0)**	**43 (100.0)**	**8 (100.0)**	**114 (100.0)**
Vomiting				
Yes	35 (55.6)	24 (55.8)	6 (75.0)	65 (57.0)
No	28 (44.4)	19 (44.2)	2 (25.0)	49 (43.0)
Total	**63 (100.0)**	**43 (100.0)**	**8 (100.0)**	**114 (100.0)**
Dehydration				
Yes	21 (33.3)	14 (32.6)	2 (25.0)	37 (32.5)
No	42 (66.7)	29 (67.4)	6 (75.0)	77 (67.5)
Total	**63 (100.0)**	**43 (100.0)**	**8 (100.0)**	**114 (100.0) **
Abdominal pain				
Yes	11 (17.5)	6 (14.0)	1 (12.5)	18 (15.8)
No	52 (82.5)	37 (86.0)	7 (87.5)	96 (84.2)
Total	**63** (**100.0**)	**43 (100.0)**	**8 (100.0)**	**114 (100.0)**

## References

[1] Hart C. A., Cunliffe N. A., Nakagomi O. D., Cook G. C., Zumla A. L. (2009). Diarrhea caused by viruses. *Manson's Tropical Diseases*.

[2] Bulanova I., Feklisova K., Titova I. (2009). Results of application of lacto-containing probiotics in viral diarrhea in young children. *Childhood Infection*.

[3] Masankova I., Begiashvili I., Shaposhnikova I. (2009). The characteristics of metabolic activity of intestinal microflora and methods of probiotics correction in viral diarrhea in children. *Russian Journal of Perinatology and Pediatrics*.

[4] Elliott E. J., Dalby-Payne J. R. (2004). Acute infectious diarrhoea and dehydration in children. *Medical Journal of Australia*.

[5] Odimayo M. S., Olanrewaju W. I., Omilabu S. A., Adegboro B. (2008). Prevalence of rotavirus-induced diarrhea among children under 5 years in Ilorin, Nigeria. *Journal of Tropical Pediatrics*.

[6] Gomwalk N. E., Gosham L. T., Umoh U. J. (1990). Rotavirus gastroenteritis in pediatric diarrhoea in Jos, Nigeria. *Journal of Tropical Pediatrics*.

[7] Audu R., Omilabu S. A., Peenze I., Steele D. (2002). Viral diarrhea in young children in two districts in Nigeria. *The Central African Journal of Medicine*.

[8] Binka F. N., Auto F. K., Odaro A. R. (2003). Incidence and risk factors of paediatric rotavirus diarrhoea in northern Ghana. *Tropical Medicine & International Health*.

[9] Gomwalk N. E., Umoh U. J., Gosham L. T., Ahmad A. A. (1993). Influence of climatic factors on rotavirus infection among children with acute gastroenteritis in Zaria, Northern Nigeria. *Journal of Tropical Pediatrics*.

[10] Abiodun P. O., Omoigberale A. (1994). Prevalence of nosocomial rotavirus infection in hospitalized children in Benin City, Nigeria. *Annals of Tropical Paediatrics*.

[11] Basu G., Rossouw J., Sebunya T. K. (2003). Prevalence of rotavirus, adenovirus and astrovirus infection in young children with gastroenteritis in Gaborone, Botswana. *East African Medical Journal*.

[12] Chung J. Y., Huh K., Kim S. W. (2006). Molecular epidemiology of human astrovirus infection in hospitalized children with acute gastroenteritis. *Korean Journal of Pediatric Gastroenterology and Nutrition*.

[13] Aminu M., Ahmad A. A., Umoh J. U., de Beer M. C., Esona M. D., Steele A. D. (2007). Adenovirus infection in children with diarrhea disease in Northwestern Nigeria. *Annals of African Medicine*.

[14] Moyo S. J., Gro N., Kirsti V. (2007). Prevalence of enteropathogenic viruses and molecular characterization of group A rotavirus among children with diarrhea in Dar es Salaam Tanzania. *BMC Public Health*.

[15] Moore P., Steele A. D., Lecatsas G., Alexander J. J. (1998). Characterization of gastroenteritis-associated adenoviruses in South Africa. *South African Medical Journal*.

[16] Ayolabi C. I., Ojo D. A., Armah G. E., Akpan I., Mafiana C. F. (2010). Detection and partial characterization of norovirus among children with acute gastroenteritis in Lagos, Nigeria. *International Journal of Medicine and Medical Sciences*.

[17] Chen C. J., Lartey B., Agbemabiese C., Mahmoud A., Armah G. (2013). The epidemiology of norovirus in Ghana: a case study of norovirus detection. *The Journal of Global Health*.

[18] Ribeiro L. R., de Oliveira Guiberti R. S., Barreira D. M. P. G. (2008). Hospitalization due to norovirus and genotypes of rotavirus in pediatric patients, state of Espírito Santo. *Memórias do Instituto Oswaldo Cruz*.

[19] Armah G. E., Gallimore C. I., Binka F. N. (2006). Characterisation of norovirus strains in rural Ghanaian children with acute diarrhoea. *Journal of Medical Virology*.

[20] Murata T., Katsushima N., Mizuta K., Muraki Y., Hongo S., Matsuzaki Y. (2007). Prolonged norovirus shedding in infants ≤6 months of age with gastroenteritis. *The Pediatric Infectious Disease Journal*.

[21] Román E., Wilhelmi I., Colomina J. (2003). Acute viral gastroenteritis: Proportion and clinical relevance of multiple infections in Spanish children. *Journal of Medical Microbiology*.

[22] Koh H., Baek S. Y., Shin J. I., Chung K. S., Jee Y. M. (2008). Co-infection of viral agents in Korean children with acute watery diarrhea. *Journal of Korean Medical Science*.

[23] Meqdam M. M., Thwiny I. R. (2007). Prevalence of group a rotavirus, enteric adenovirus, norovirus and astrovirus infections among children with acute gastroenteritis in Al-Qassim, Saudi Arabia. *Pakistan Journal of Medical Sciences*.

[24] Najafi A., Shafiei H., Barazesh A. (2011). Epidemiological and clinical characteristics of gastroenteritis associated with enteric adenovirus in hospitalized children in Bushehr Province, Iran. *African Journal of Microbiology Research*.

[25] Najafi A., Iranpour D., Najafi S. (2013). Epidemiological surveillance of norovirus diarrhea in hospitalized children with acute gastroenteritis in south of Iran. *Jundishapur Journal of Microbiology*.

[26] Bresee J. S., Widdowson M.-A., Monroe S. S., Glass R. I. (2002). Foodborne viral gastroenteritis: challenges and opportunities. *Clinical Infectious Diseases*.

[27] Staat M. A., Azimi P. H., Berke T. (2002). Clinical presentations of rotavirus infection among hospitalized children. *Pediatric Infectious Disease Journal*.

[28] Dey S. K., Shimizu H., Phan T. G. (2009). Molecular epidemiology of adenovirus infection among infants and children with acute gastroenteritis in Dhaka City, Bangladesh. *Infection, Genetics and Evolution*.

[29] Hamkar R., Yahyapour Y., Noroozi M. (2010). Prevalence of rotavirus, adenovirus, and astrovirus infections among patients with acute gastroenteritis in, Northern Iran. *Iranian Journal of Public Health*.

[30] Jakab F., Németh V., Oldal M. (2010). Epidemiological and clinical characterization of norovirus infections among hospitalized children in Baranya County, Hungary. *Journal of Clinical Virology*.

[31] Junquera C. G., de Baranda C. S., Mialdea O. G., Serrano E. B., Sánchez-Fauquier A. (2009). Prevalence and clinical characteristics of norovirus gastroenteritis among hospitalized children in Spain. *Pediatric Infectious Disease Journal*.

